# Enhanced Vascular-like Network Formation of Encapsulated HUVECs and ADSCs Coculture in Growth Factors Conjugated GelMA Hydrogels

**DOI:** 10.1021/acsbiomaterials.4c00465

**Published:** 2024-04-18

**Authors:** Sasinan Bupphathong, Joshua Lim, Hsu-Wei Fang, Hsuan-Ya Tao, Chen-En Yeh, Tian-An Ku, Wei Huang, Ting-Yu Kuo, Chih-Hsin Lin

**Affiliations:** † Graduate Institute of Nanomedicine and Medical Engineering, College of Biomedical Engineering, Taipei Medical University, Taipei 110, Taiwan; ‡ High-Value Biomaterials Research and Commercialization Center, National Taipei University of Technology, Taipei 10608, Taiwan; § Department of Chemical Engineering and Biotechnology, National Taipei University of Technology, Taipei 10608, Taiwan; ∥ Institute of Biomedical Engineering and Nanomedicine, National Health Research Institutes, Zhunan 35053, Taiwan; ⊥ School of Biomedical Engineering, College of Biomedical Engineering, Taipei Medical University, Taipei 110, Taiwan; # Department of Orthodontics, Rutgers School of Dental Medicine, Newark, New Jersey 07103, United States

**Keywords:** *gelMA*, *vEGF*, *bFGF*, *vascular formation*, *hUVECs*, *hADSCs*

## Abstract

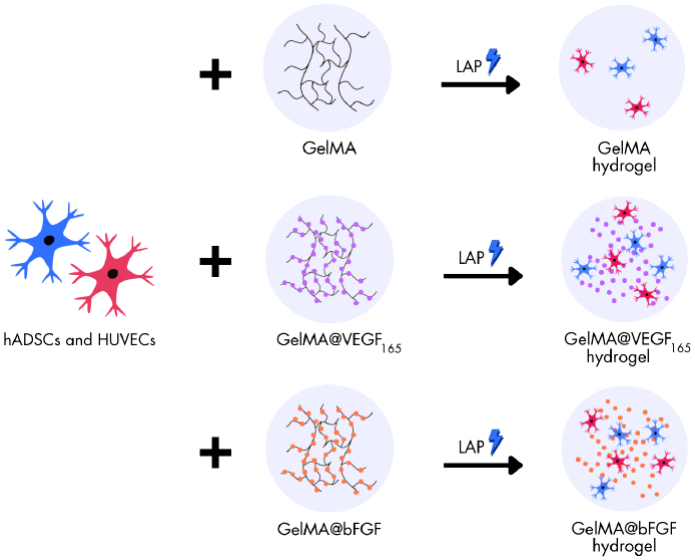

Tissue engineering primarily aimed to alleviate the insufficiency of organ donations worldwide. Nonetheless, the survival of the engineered tissue is often compromised due to the complexity of the natural organ architectures, especially the vascular system inside the organ, which allows food-waste transfer. Thus, vascularization within the engineered tissue is of paramount importance. A critical aspect of this endeavor is the ability to replicate the intricacies of the extracellular matrix and promote the formation of functional vascular networks within engineered constructs. In this study, human adipose-derived stem cells (hADSCs) and human umbilical vein endothelial cells (HUVECs) were cocultured in different types of gelatin methacrylate (GelMA). In brief, pro-angiogenic signaling growth factors (GFs), vascular endothelial growth factor (VEGF_165_) and basic fibroblast growth factor (bFGF), were conjugated onto GelMA via an EDC/NHS coupling reaction. The GelMA hydrogels conjugated with VEGF_165_ (GelMA@VEGF_165_) and bFGF (GelMA@bFGF) showed marginal changes in the chemical and physical characteristics of the GelMA hydrogels. Moreover, the conjugation of these growth factors demonstrated improved cell viability and cell proliferation within the hydrogel construct. Additionally, vascular-like network formation was observed predominantly on GelMA@GrowthFactor (GelMA@GF) hydrogels, particularly on GelMA@bFGF. This study suggests that growth factor-conjugated GelMA hydrogels would be a promising biomaterial for 3D vascular tissue engineering.

## 1 Introduction

GelMA hydrogel has emerged as a promising material in the field of biomedical research due to its biocompatibility, degradability, and the ability to modify its chemical and mechanical properties as needed.^1−3^ The methacryloyl groups present in GelMA enable photo-cross-linking, which allows for the formation of three-dimensional constructs with a high degree of control. Moreover, GelMA inherits the arginylglycylaspartic acid (RGD) tripeptide from gelatin, which assists in several cellular activities, including attachment, spreading, and differentiation. It also contains matrix metalloproteinase (MMP) sequences that promote enzymatic degradation and wound healing.^4^ While GelMA presents considerable promise in tissue engineering, addressing the challenge of providing adequate vascularization would demand equal attention. This is because these engineered matrices frequently create a diffusion barrier which restricts the flow of nutrients and oxygen, thereby posing greater risks of starvation and hypoxia for cells situated at the center of the construct.^5−7^ Thus, experts recommend that engineered constructs should be situated within a distance of 200 μm from blood vessels.^8,9^ This limitation is particularly critical for larger tissue constructs, as the mass transport requirement is amplified.^10,11^ To address these challenges, previous studies proposed incorporating growth factors and pro-angiogenic signaling factors into engineered scaffolds to establish a more natural environment that can direct cell behavior and modulate cell proliferation, differentiation, and tissue regeneration.^12,13^ To date, there are numerous angiogenic growth factors that have been identified, among which the most prominent are VEGF and bFGF.^14^

VEGF is a critical regulatory protein that plays a pivotal role in promoting the formation of blood vessels. It has been widely employed in conjunction with hydrogels, showcasing its ability to induce cell migration, tube formation, and *in vivo* angiogenesis.^15,16^ A previous research conducted by Jang et al. showed the advantages of incorporating growth factors into GelMA hydrogel for skin tissue engineering.^17^ Specifically, their finding revealed that directly inoculating VEGF-mimicking peptides to GelMA bioink and employing 3D bioprint technology to implant them in the dermis of pig models led to a significantly higher expression of CD31 and α-SMA compared to the standard DuoDerm and GelMA hydrogel patch groups. However, Prakash Parthiban et al. utilized an alternative approach by creating cell-laden GelMA hydrogels that were covalently linked to VEGF-mimicking peptides through UV irradiation.^18^ The developed GelMA + VEGF mimicking peptide demonstrated its capability in promoting vascularization, as evidenced by the higher levels of CD34, Angiopoietin-2, and von Willebrand factor expressed by the HUVECs in a 3D culture. It is widely considered that bFGF is just as essential as VEGF, and it can promote the growth of blood vessels during the healing of tissue and increase VEGF expression in vascular smooth muscle cells.^19,20^ As of our knowledge, no one has attempted to immobilize FGF onto gelatin or collagen scaffolds using EDC/NHS coupling. However, immobilization of FGF by direct immersion has previously been attempted with electrospun PCL–gelatin fibrous membranes^21^ and polydopamine-coated poly(xylitol dodecanedioic acid) films.^22^ Direct inoculation, nonetheless, has provided positive outcomes. Luo et al. developed a nerve conduit using a composite membrane composed of cellulose and soy protein isolate (CSM), which was filled with bioactive GelMA hydrogels containing recombinant human basic fibroblast growth factor (GFD) and dental pulp stem cells (DPSCs). In a rat model with a 15 mm long sciatic nerve defect, implantation of the CSM-GFD conduits promoted significant axonal regeneration, remyelination, and functional recovery comparable to nerve autografts and the DPSCs directly differentiated into new nerve tissue at the defect site.^23^

The present study reports the synthesis and characterization of GelMA, GelMA@VEGF_165_, and GelMA@bFGF hydrogels, followed by the encapsulation of HUVECs and hADSCs within each hydrogel type using photo-cross-linking with blue light irradiation. Subsequently, the growth, morphology, and protein marker expression of the encapsulated cells were assessed. Our results indicate that GelMA@bFGF may be an appropriate biomaterial for the development of three-dimensional vascular tissue engineering.

## 2 Materials and Methods

### 2.1 GelMA Synthesis

GelMA was synthesized according to a previously reported method.^24^ In brief, a 10% (w/v) gelatin solution was prepared by dissolving 10 g of porcine skin gelatin (type A, 300 Bloom; Sigma-Aldrich, USA) in 100 mL of 0.25 M carbonate-bicarbonate (CB) buffer. The pH was then adjusted to 9.4, and the solution was stirred at 50–55 °C for at least 1 h or until the gelatin was completely dissolved. Subsequently, 0.938 mL of methacrylic anhydride (MAA, 94%; Sigma-Aldrich, USA) was carefully added to the gelatin solution, while maintaining continuous stirring for another hour. To stop the reaction, the pH was adjusted to 7.4. The solution was then dialyzed (6–8 kDa MWCO cellulose dialysis membrane, Biomate, Taiwan) against deionized water at 40 °C for 5 days and lyophilized for an additional 5 days to yield an oyster-white solid product. Finally, GelMA was stored at −20 °C until use.

### 2.2 Quantification of Degree of Substitution

The degree of methacrylation was measured using the trinitrobenzenesulfonic acid (TNBS) assay, a method previously developed by Habeeb.^25^ Briefly, lyophilized GelMA was dissolved in 200 μg/mL (w/v) of 0.1 M sodium bicarbonate (pH8.5). Then, 0.5 mL of 0.01% (w/v) TNBS solution was added to 0.5 mL of each sample solution, and the samples were incubated at 37 °C for 2 h. To stop and stabilize the reaction, 0.5 mL of 10% sodium dodecyl sulfate (SDS) and 0.25 mL of 1 M hydrogen chloride (HCl) were added to each sample. The optical density was determined using a microplate spectrophotometer (Epoch2, Biotek Synergy, USA) at 335 nm. The extent of substitution was calculated by comparing the amount of free amino groups remaining in GelMA, and the degree of methacrylation was calculated as follows:
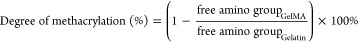
1

The modified free amino group in GelMA was calculated by subtracting the reference free amino acid group in gelatin with the remaining free amino acid group in GelMA. A standard curve was established using varying concentrations of alanine, a compound with a chemical structure similar to gelatin. Four replicates of GelMA synthesis were performed to confirm the degree of methacrylation of GelMA.

### 2.3 Conjugation of VEGF and FGF on GelMA

The conjugation of VEGF_165_ and bFGF with GelMA was accomplished using the EDC/NHS chemical coupling method. Briefly, 300 mg of GelMA was dissolved in 5 mL of 1× PBS. Then, 0.5 mg of *N*-(3-(dimethylamino)propyl)-*N*0-ethylcarbodiimide hydrochloride (EDC) (Sigma-Aldrich, USA) and 0.5 mg of *N*-hydroxysuccinimide (NHS) (Sigma-Aldrich, USA) were added and agitated at 125 rpm. After activating the GelMA hydrogel for 30 min, we utilized 1 μg of growth factors (VEGF165 or bFGF) per 300 mg of GelMA to achieve a solution concentration of 0.2 μg/mL for the growth factors during the synthesis process. The mixture was then left to stir for 6 h at 27 °C in order to facilitate the coupling reaction and the binding of the growth factors within the GelMA hydrogel. The GelMA@VEGF_165_ and GelMA@bFGF solutions were subsequently subjected to dialysis against deionized water at room temperature to remove unbound growth factors and impurities for a period of 5 days, followed by freeze-drying for an additional 5 days. Finally, the GelMA@GF sponges were stored at −20 °C until use.

### 2.4 Chemical Characterization and Quantification of Conjugated GFs in GelMA

#### 2.4.1 ^1^H Nuclear Magnetic Resonance (^1^H NMR)

To verify the successful methacrylation of gelatin in GelMA and the intactness of the methacryloyl group on GelMA@GFs, the chemical structures of GelMA, GelMA@VEGF_165_, and GelMA@bFGF were characterized using Agilent DD2 600 MHz NMR. The^1^H NMR spectra were acquired by dissolving GelMA, GelMA@VEGF_165_, and GelMA@bFGF at a concentration of 8 mg/mL in deuterium oxide (D_2_O). All measurements were done at 40 °C, and the D_2_O peak was identified at 4.671 ppm.

#### 2.4.2 Quantification of Conjugated Growth Factors

The levels of conjugated VEGF_165_ and bFGF in GelMA were quantified using ELISA kits according to the manufacturer’s protocols specific for VEGF_165_ (MyBioSource, Canada) and bFGF (Bioss Antibodies, USA), respectively. In brief, the growth factor standard curves were established through the use of serial dilutions of standard samples provided within the kit. To prepare the GelMA@GF samples, GelMA@VEGF_165_ and GelMA@bFGF were dissolved at 0.1 mg/mL in the standard diluent buffer for the ELISA colorimetric assays. The absorbances at 450 nm were used to plot the standard curve, and the equations derived from the standard curves were used to determine the amount of growth factors in the samples.

### 2.5 Physical Characterization of GelMA and GelMA@GF Hydrogel

In the course of investigating the physical characterization of GelMA and GelMA@GF hydrogels, the samples were initially subjected to photo-cross-linking. In brief, 1% w/v lithium phenyl (2,4,6-trimethylbenzoyl)^7^ photoinitiator was first completely prepared and dissolved in 1× PBS buffer. Subsequently, dissolution of GelMA, GelMA@VEGF_165_, and GelMA@bFGF was carried out with 1× PBS buffer. Thereafter, the prepared 1% LAP solution was added to attain a final LAP concentration of 0.08% (w/v) and final GelMA concentrations of 10%, 15%, or 20% (w/v). Finally, the hydrogel was prepared in a cylindrical polytetrafluoroethylene mold (Ø = 8 mm, height = 1 mm) and irradiated with 405 nm blue light for 30 s.

#### 2.5.1 Morphological Analysis

Cross-linked GelMA and GelMA@GF hydrogel samples at different concentrations were lyophilized and then coated with gold using a sputter coater (SC7620, Quorum Technologies, UK). A scanning electron microscope (SEM) (Hitachi SU-3500 SEM, Japan) was used to take images of the GelMA samples. Pore segmentation was performed using MorphoLibJ plugin followed by Analyze Particles function in Fiji.^26,27^ Average pore size radii were obtained from the particle analysis and then were calculated using the following equation:
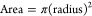
2

#### 2.5.2 Mechanical Properties of GelMA and GelMA@GFs

Oscillatory frequency-sweep measurements on the GelMA and GelMA@GF hydrogels were performed using a rheometer (MCR302 Anton-Paar, Taiwan) equipped with a 10 mm parallel plate measuring system to determine the storage modulus (*G*′) and loss modulus (*G*″). The storage modulus was measured at 0.1% strain and 0.1–10 Hz within the viscoelastic range, with the running temperature maintained at 37 °C throughout the measurements.

#### 2.5.3 Swelling Profile of GelMA and GelMA-GFs

The measurement of swelling profiles involving the weighing of GelMA hydrogels was carried out at room temperature. Briefly, various concentrations (10%, 15%, and 20%) of lyophilized GelMA, GelMA@VEGF_165_, and GelMA@bFGF samples were weighed and immersed in 1× distilled water (ddH_2_O). Then, the samples were gently taken out, blotted with filter paper to eliminate surface water, and weighed until the hydrated samples attained a constant value. The average weight of five samples were recorded at different time points, and the swelling degree was defined as follows:
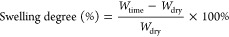
3

where *W*_time_ represents the mass of the GelMA hydrogels at each time point (5, 15, 30, 60, 120, 180, and 240 min) being measured and *W*_dry_ denotes their lyophilized weight.

#### 2.5.4 Enzymatic Degradation Profile of GelMA and GelMA@GFs

GelMA, GelMA@VEGF_165_, and GelMA@bFGF hydrogels (10%, 15%, and 20%) were tested for enzymatic degradation. In brief, hydrogel constructs of 100 μL of GelMA were submerged in 1 mL of 0.05% (w/v) collagenase (125 CDU/mg) with 3 mM CaCl_2_ in 1× PBS and kept at 37 °C. The weights of the hydrogels were measured at different time points, and the mass loss of the GelMA hydrogels was also measured using the following equation:
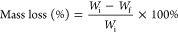
4

where *W*_f_ represents the weight of each GelMA hydrogel at the specific time point (30, 60 90, 120, 180, 240, 300, 360, 420, and 480 min) being measured and *W*_i_ denotes the initial weight of each GelMA hydrogel after photo-cross-linking.

### 2.6 Cell Culture

HUVECs were obtained from Bioresource Collection and Research Center (BCRC, Taiwan) and cultured in complete M199 medium, which contains 90% Medium 199 (Gibco, USA) with 25 U/mL heparin (Sigma-Aldrich, USA), 30 ug/mL endothelial cell growth supplement (Merck, Germany), 2.2 g/L sodium bicarbonate (Sigma-Aldrich, USA), 10% fetal bovine serum (FBS, Peak, USA), and 100 U/mL Penicillin/Streptomycin (Gibco, USA). All experiments were performed with HUVECs between passages 5 to 14. hADSCs were sourced from POIETICS and were cultured in complete ADSC medium (ADSC Growth Media BulletKit, Lonza, Switzerland). All cell cultures were maintained at 37 °C with 5% CO_2_, and the media were changed every 2 days. Cells were subcultured when the confluency reached 75–85%. A coculture medium for the HUVECs and hADSCs was also prepared by combining a mixture of complete M199 medium and complete ADSCs medium at different percentages (Table 1). Assessment of cell proliferation is accomplished through utilization of the CCK-8 assay in conventional 96-well plates over a period of 7 days, followed by measurement of absorbance at 450 nm.

**Table 1:** Different Culture Media Percentage

media	complete M199 media	complete ADSC media
Media A	100	0
Media B	75	25
Media C	50	50
Media D	25	75
Media E	0	100

### 2.7 In Vitro Biocompatibility of Encapsulated HUVECs and hADSCs in GelMA

To fabricate GelMA@GF hydrogels, 1% w/v Lithium Phenyl (2,4,6-Trimethylbenzoyl)^7^ photoinitiator was first completely prepared and dissolved in 1× DPBS buffer. Sponge GelMA, GelMA@VEGF_165_, and GelMA@bFGF were then dissolved in 1× DPBS. Sterilization of hydrogel was conducted by squeezing the GelMA hydrogels against a 0.22 μm syringe filter. Thereafter, hADSCs (7.5 × 10^4^ cells/mL) and HUVECs (7.5 × 10^4^ cells/mL) were added to the GelMA solution. Subsequently, the previously dissolved LAP solution was added to achieve a final concentration of 0.08% LAP in the total solutions. Finally, 30 μL of cell-laden hydrogels were prepared in a stainless-steel mold (Ø = 6.5 mm, height = 1 mm) and irradiated with 405 nm blue light for 15 s.

#### 2.7.1 Cell Viability/Cytotoxicity

The LIVE/DEAD cell staining technique was performed using a commercial kit (Thermo Fisher Sciences, USA) to assess the viability of cells encapsulated within GelMA and GelMA@GF hydrogels under three different media conditions: (i) complete M199 medium (M199), (ii) complete ADSC medium (ADSC), and (iii) 50:50 (M199:ADSC) medium. The GelMA and GelMA@GF hydrogels were prepared in a 24-well plate and placed in a humidified incubator (37 °C, 5% CO_2_) for 14 days. The media were changed every 48–72 h. Following this period, the GelMA hydrogels were washed twice with DPBS and then stained with a LIVE/DEAD solution according to the manufacturer’s instructions. The samples were then observed using a laser scanning confocal microscope (TCS SP5 Confocal Spectral Microscope Imaging System, Leica, Germany) (a slice width of 4.99 um). The live cells were labeled with calcein-AM, which emitted green fluorescence, while the dead cells were labeled with BOBO-3 iodide and emitted red fluorescence. Image analysis software, ImageJ, was utilized to quantify the cell viability of the encapsulated cells within the culture media.

#### 2.7.2 Cell Morphology and 3D Organization

Prior to immunofluorescence staining, the cell-laden hydrogels were rinsed with cold 1× PBS thrice for 5 min each rinse on the rocking platform. Next, the samples were fixed with a 10% formalin solution and permeabilized with 0.3% Triton X-100 at room temperature for 30 min. The samples were then blocked with 10% FBS in 1× PBS for one hour at room temperature. Primary antibodies including mouse-antihuman CD31 (PECAM-1) (Invitrogen, USA) (1:100) rabbit-antihuman α-smooth muscle actin (α-SMA) (Proteintech, USA) (1:100), and donkey-antihuman CD90 (Biotechne/R&D Systems, USA) (1:50) were incubated with the samples overnight at 4 °C. The hydrogels were then washed with 1% FBS in 1× PBS thrice. Then, the samples were incubated with fluorescent-labeled secondary antibodies Goat anti-Mouse Alexa FluorTM 488 (1:500), Donkey anti-Rabbit Alexa Fluor 555 (1:500), and Donkey anti-Sheep Alexa Fluor 633 (1:500) for one hour at room temperature in the dark. Hoechst 33342 (1:1500) was added to stain the nuclei. After the last washing process, the stained hydrogels were preserved in 1× PBS and images were captured using a laser scanning confocal microscope (magnification = 10×, z slice width = 4.99 μm).

### 2.8 Statistical Analysis

The data presented in this study are expressed as the mean ± standard deviation (SD). Statistical significance was determined using the Student’s *t* test and denoted by asterisks (*p < 0.05, **p < 0.01, and ***p < 0.001) to indicate the level of statistical significance.

## 3 Result and Discussion

### 3.1 Synthesis and Chemical Characterization of GelMA and GelMA@GFs

The extent of methacrylation in GelMA has a direct influence on the material’s properties and performance.^28,29^ The TNBS assay was employed to evaluate the degree of methacrylation in GelMA by quantifying the remaining amino groups after the chemical reaction between methacrylic anhydride and primary amines on gelatin.^29^ As exhibited in Table 2, the average substitution percentage from 4 produced batches adapted from Zhu et al.’s protocol^24^ is approximately 97%. Additionally, the NMR spectrum of GelMA (Figure S1) has proven the success of GelMA synthesis.

**Table 2:** Summary of Degree of Methacrylation of Various GelMA Synthesis Batches (TNBS assay)

batch number	degree of substitution (%)
I	98.06%
II	99.22%
III	94.00%
IV	96.71%

Applying NHS/EDC coupling reaction to attach growth factors to biomaterials was proposed by Shen et al. when they linked mouse recombinant VEGF_165_ with collagen in order to address the challenge of stimulating the invasion and proliferation of endothelial cells into scaffolds, aiming to create functional vascular networks within engineered tissues.^30^ This approach was later adapted by Byambaa et al., who implemented a modified method for chemically conjugating VEGF to GelMA, which involved reacting GelMA first with succinic anhydride to convert its amine groups into carboxylic acid groups. This modification eliminated potential side reactions resulting from remaining lysine residues.^31^ Their study demonstrated that binding VEGF to polymers, particularly GelMA, resulted in a slower release profile as opposed to directly mixing it into the hydrogel, which consequently contributed to the enhancement of vascularization in the bone scaffold. Employing the same principles, we conjugated human VEGF_165_ and bFGF onto our GelMA through EDC/NHS coupling. The successful conjugation of VEGF_165_ and bFGF with GelMA was confirmed and quantified with VEGF_165_ and bFGF ELISA kits because the conjugation of growth factors on GelMA cannot be detected by common molecular characterization techniques such as ^1^H NMR (Figure S2). As shown in Figure S2A,B, the ELISA assay results showed that there was no false-positive detection, as the growth factors were not detectable in GelMA samples. Meanwhile, the quantitative results of VEGF_165_ and bFGF conjugated onto GelMA were 285.54 and 346.20 pg/mg, respectively (Figure S2C).

### 3.2 Microstructural and Mechanical Analysis of GelMA and GelMA@GFs

**Figure 1:**
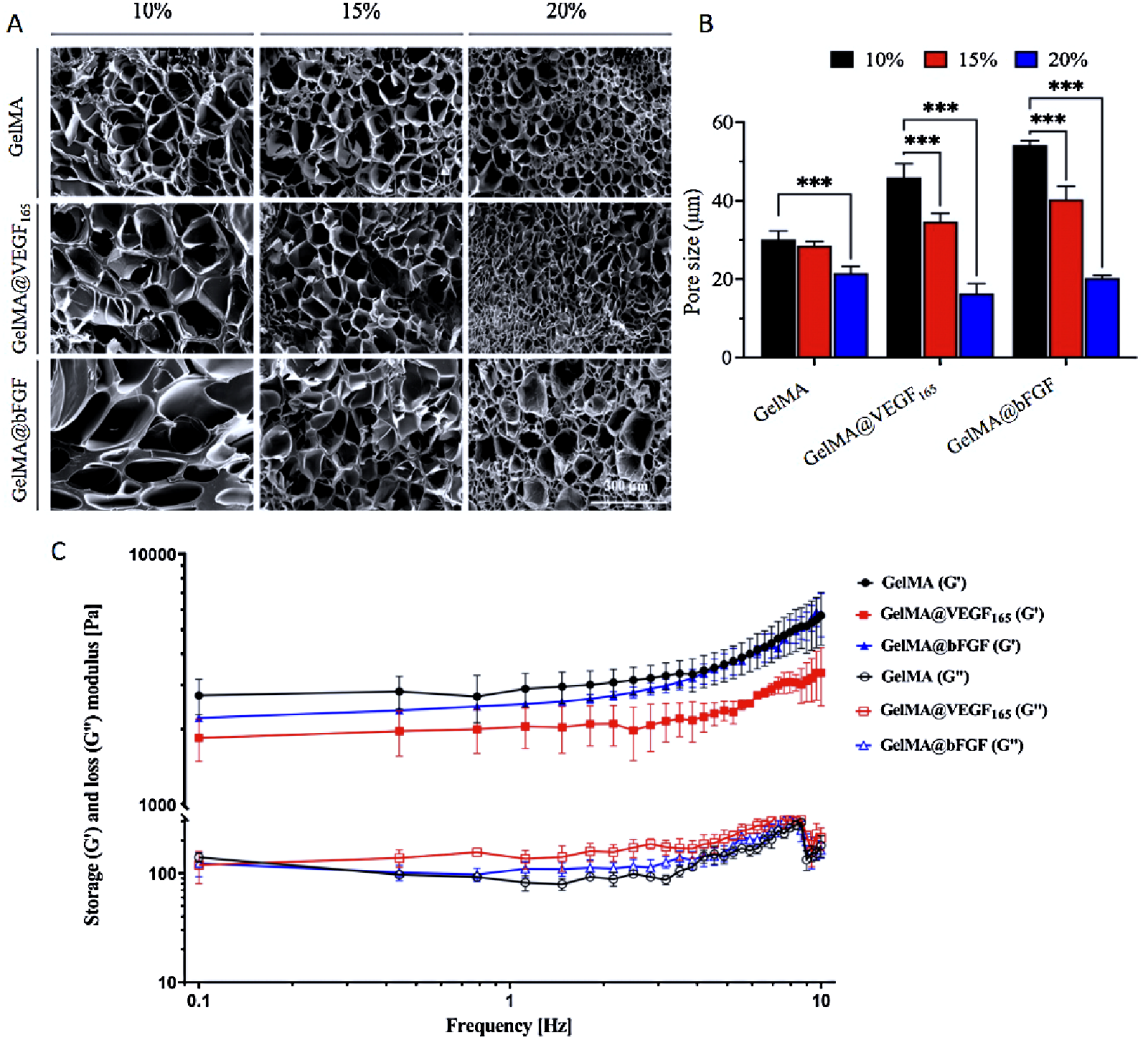
(A) SEM micrographs of different type of GelMAs at different concentrations. (B) Pore-sized analysis result of different type of GelMAs at different content ratios based on SEM micrographs. (C) Oscillatory frequency sweep of 15% GelMA, GelMA@VEGF_165_, and GelMA@bFGF hydrogels.

Cross-sections of GelMA hydrogels at various concentrations display porous structures, allowing for nutrient transport and cell proliferation.^32^ The SEM images in Figure 1A demonstrate that lyophilized GelMA, GelMA@VEGF_165_, and GelMA@bFGF hydrogels exhibit porous morphology. The pore size analysis result in Figure 1B shows that the pore size significantly decreases when the concentration of GelMA increases. Additionally, among three types of GelMA at the same prepared concentration (10% and 15%), the growth factor modification on GelMA causes a larger pore size within the hydrogel structure. The bFGF coupling results in the largest pore size, followed by the modification with VEGF_165_, and the unmodified GelMA displayed the smallest pore area. It is possible that the bigger pore size in the modified GelMA could come from the volume of the growth factor that hinders the cross-linking degree and leads to the larger pore. However, the difference in pore size at 20% concentration does not follow this trend. As mentioned in other studies, the cross-linking density in GelMA constructs influences the mechanical properties of the hydrogel.^33,34^ The measurement of viscoelastic properties encompasses two components: the storage modulus (*G*′), which signifies the elastic and reversible response of the material, and the loss modulus (*G*″), which denotes the viscous and irreversible rearrangement of its polymeric structure. This study conducted a rheological examination of GelMA, GelMA@VEGF_165_, and GelMA@bFGF to assess their viscoelastic properties using oscillatory rheometry. As shown in Figure 1C, at the concentration of 15%, both *G*′ and *G*″ of each type of GelMA share the similarity in viscoelasticity.

**Figure 2:**
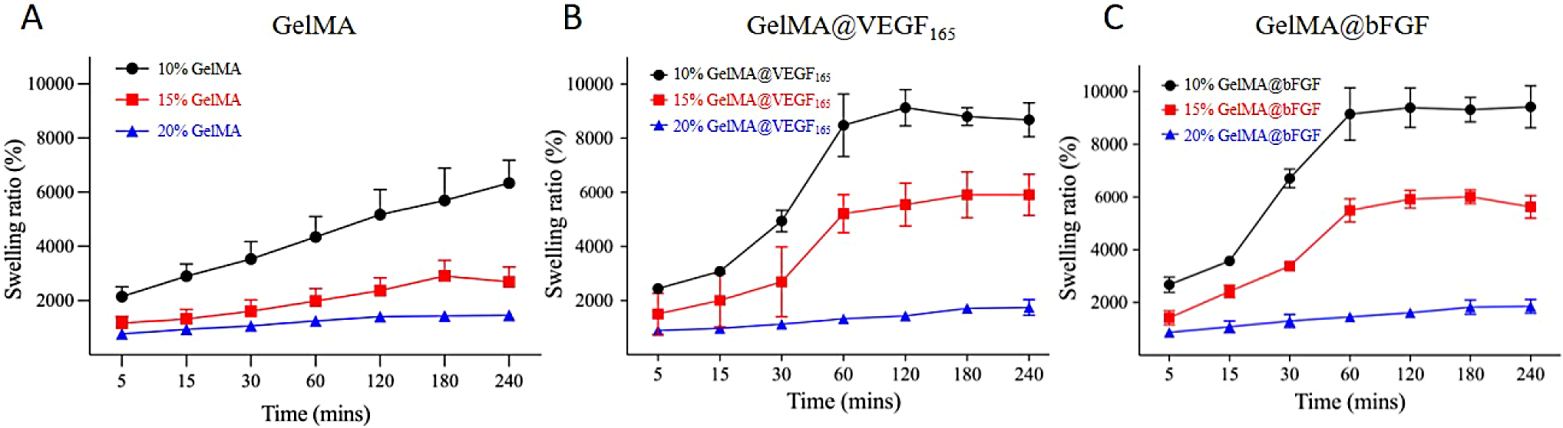
Swelling behaviors of (A) GelMA, (B) GelMA@VEGF_165_, and (C) GelMA@bFGF hydrogels in ddH_2_O.

The hydrogels’ swelling capacity significantly influences gas exchange, fluid absorption, nutrient transfer, and cell encapsulation.^35,36^ Understanding the swelling behavior of hydrogels is essential for accurately predicting nutrient and waste diffusion in both cell cultures and drug delivery systems. This behavior indicates the hydrogel’s capacity to absorb fluid, which is critical for closely mimicking the physiological environment of tissues. The swelling ratios of GelMA and GelMA@GF hydrogels with 10%, 15%, and 20% concentrations were assessed at various time intervals. As displayed in Figure 2, the swelling ability is inversely proportional to the concentration in all types of GelMA. Specifically, at 15%, the growth factor-conjugated GelMA can absorb water approximately 6000% of the dry weight of the hydrogel, while the maximum water absorption ability of unmodified GelMA is reported to be slightly below 3000% of the dry weight. Moreover, the water retention in GelMA@GF hydrogels reach their plateaus within 1 day, while that of unmodified GelMA takes 2 days to reach a stable level (Figure S3).

**Figure 3:**
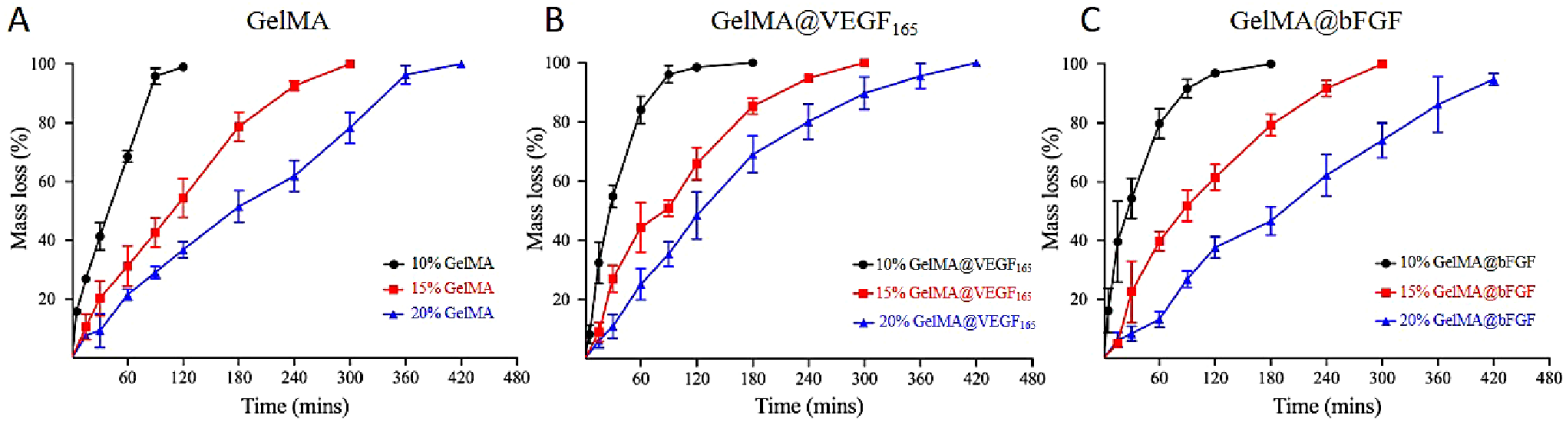
Enzymatic degradation of (A) GelMA, (B) GelMA@VEGF_165_, and (C) GelMA@bFGF hydrogels in collagenase-CaCl_2_ contained in PBS.

Scaffolds serve as templates for the growth of new tissue. In order to facilitate this process, the materials should be biodegradable so it can be broken down by the body’s cells during neo-tissue genesis.^37^ GelMA hydrogels contain the MMP-sensitive sites, which allow encapsulated cells in GelMA hydrogels to degrade and remodel the surrounding hydrogel using cell-secreted extracellular matrix (ECM).^38^ In order to study the degradation behavior of GelMA, samples were immersed in collagenase and underwent accelerated enzymatic degradation. Figure 3 shows that the higher concentration of GelMA requires a longer time for the enzymatic degradation process, regardless of the presence of growth factors. From the data gathered, we can infer that having a higher concentration of GelMA allows a higher degree of chemical cross-linking, which leads to a smaller pore size, lower water absorbability, and slower enzymatic degradability. Similar to previous studies, our data demonstrated the direct correlation between the concentration of GelMA and its physical properties.^39,40^

### 3.3 Biocompatibility and Cell Growth Profile of HUVECs and hADSCs in GelMA and GelMA@GFs

Different types of cells require different types of culture media. In the more complex culture system like coculturing, culture media optimization is necessary. In this work, five types of culture media were evaluated for the optimal growth of the HUVECs and hADSCs coculture. The cell proliferation of 2D coculture shown in Figure S4 reveals that a 50:50 ratio of complete M199 and complete ADSC medium is the most favorable in promoting cell growth. Therefore, this ratio was chosen in subsequent experiments.

Compared to 2D flat cell cultures, 3D hydrogel cultures reflect the physiological state of cells in the *in vivo* environment more accurately.^41^ One of the challenges in creating a 3D environment with photo-cross-linkable polymers, such as GelMA, is ensuring minimal cytotoxicity from the photoinitiators.^42^ To address this, we investigated the impact of various concentrations of LAP in GelMA. The cell viability of HUVECs after LAP treatment is shown in Figure S5. The results show that the presence of LAP from 0.05% to 0.1% (w/v) with 405 nm light irradiation does not compromise the cell viability of HUVECs. In this study, 0.08% (w/v) LAP was used, as this concentration allowed the cross-linking time of 30 s for a 1-mm-thick GelMA hydrogel. Additionally, when combining GelMAs with 0.08% (w/v) LAP, both unmodified GelMA and GelMA@GFs at 15% show improvement of cell viability (Figure S6). Consequently, 15% GelMA and GelMA@GFs were utilized throughout this study.

**Figure 4:**
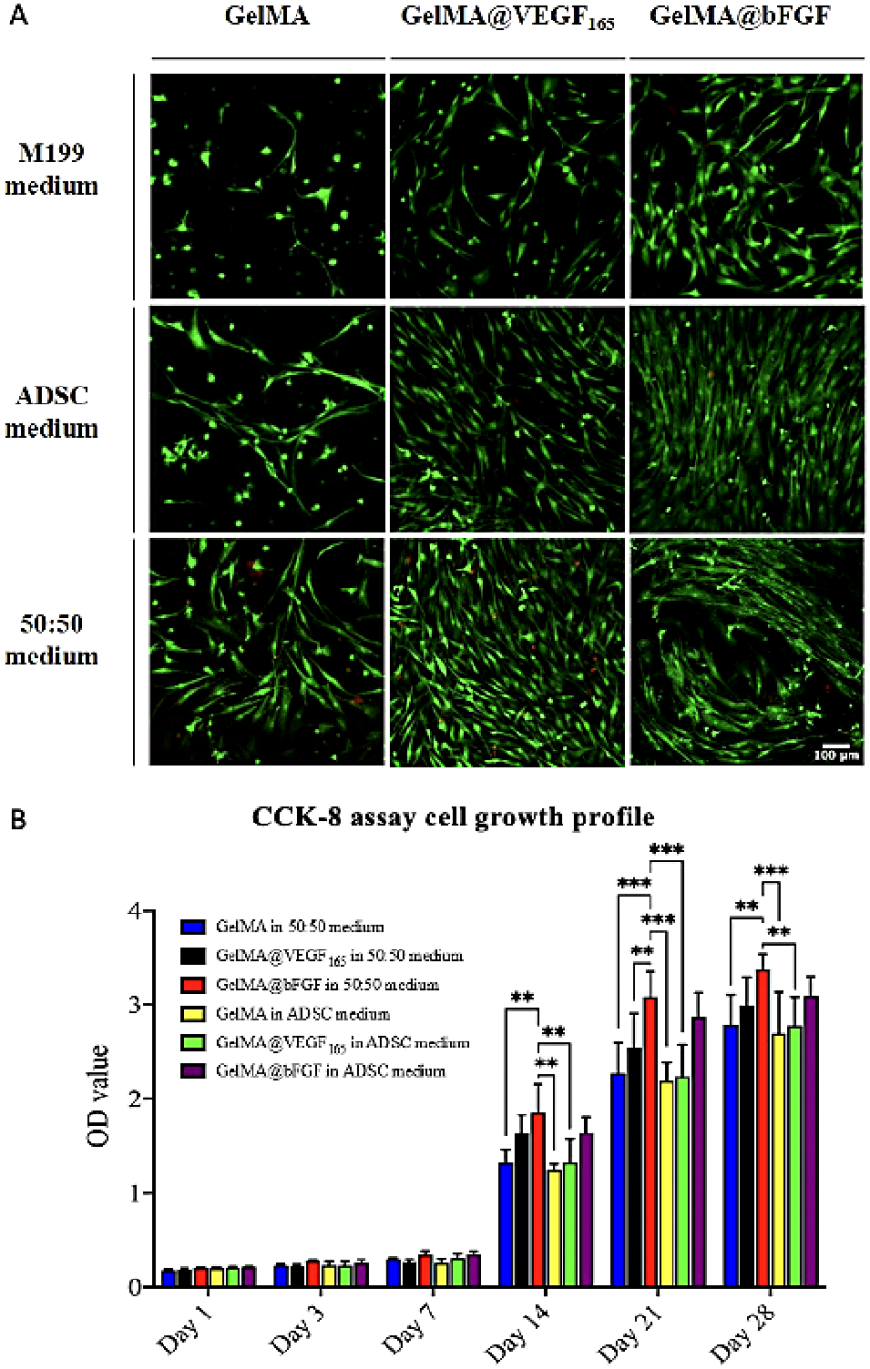
Encapsulated HUVECs and hADSCs in 3D hydrogels. (A) Day 14 LIVE/DEAD imaging of encapsulated cells. The images are displayed in Z-projection and merged channels (Green: live cells, red: dead cells, blue: nuclei, scale bar: 100 μm). (B) CCK-8 assay cell growth profile of GelMA, GelMA@VEGF_165_, and VEGF@bFGF in days 1, 3, 7, 14, and 28 of coculture in complete ADSC and complete M199:ADSC (50:50) medium.

The biocompatibility of GelMA and GelMA@GFs for cell encapsulation was assessed using LIVE/DEAD staining. After 14 days of culture, GelMA@VEGF_165_ and GelMA@bFGF hydrogels exhibited robust cellular viability (Figures 4, S7 and S8). The results from the cell proliferation assay conducted in the 3D culture were consistent with those obtained in the 2D culture. Specifically, the M199:ADSC medium at 50:50 ratio proved to be the most favorable condition, while the 100% complete M199 medium was found to be the least conducive for cell growth in the coculture system. Between the GelMA@GFs, cells encapsulated in GelMA@bFGF demonstrated higher levels of cellular proliferation in both complete ADSC and 50:50 medium compared to those encapsulated in GelMA@VEGF_165_.

### 3.4 Vasculogenic Formation of Encapsulated HUVECs and hADSCs in GelMA and GelMA@GF Hydrogels

Ensuring the establishment of functional blood vessel networks is crucial for determining the successful integration and regeneration of engineered constructs in host tissue.^43^ Undeniably, GelMA’s biocompatibility and tunable physical properties have established itself as a promising material for promoting tissue vascularization.^44,45^ Previously, we demonstrated that modifying the mechanical properties of gelatin methacrylate can facilitate the differentiation of mesenchymal stem cells into endothelial and osteogenic cells, as well as the subsequent formation of capillary-like networks.^46^ In this study, VEGF_165_ and bFGF which were reported to help promote vasculogenesis were chosen to be immobilized onto GelMA, with the aim of facilitating vascularization in tissue-engineered constructs. hADSCs were also cocultured with HUVECs in the 50:50 (M199:ADSC) medium as they possess the ability to promote vascularization of HUVECs and are capable of differentiating into endothelial cells or supporting cells, such as pericytes.^47−49^ Additionally, it has been proposed that ADSCs secrete angiogenic molecules; however, for the sustenance or advancement of the network, these molecules alone may not be sufficient.^50^

**Figure 5:**
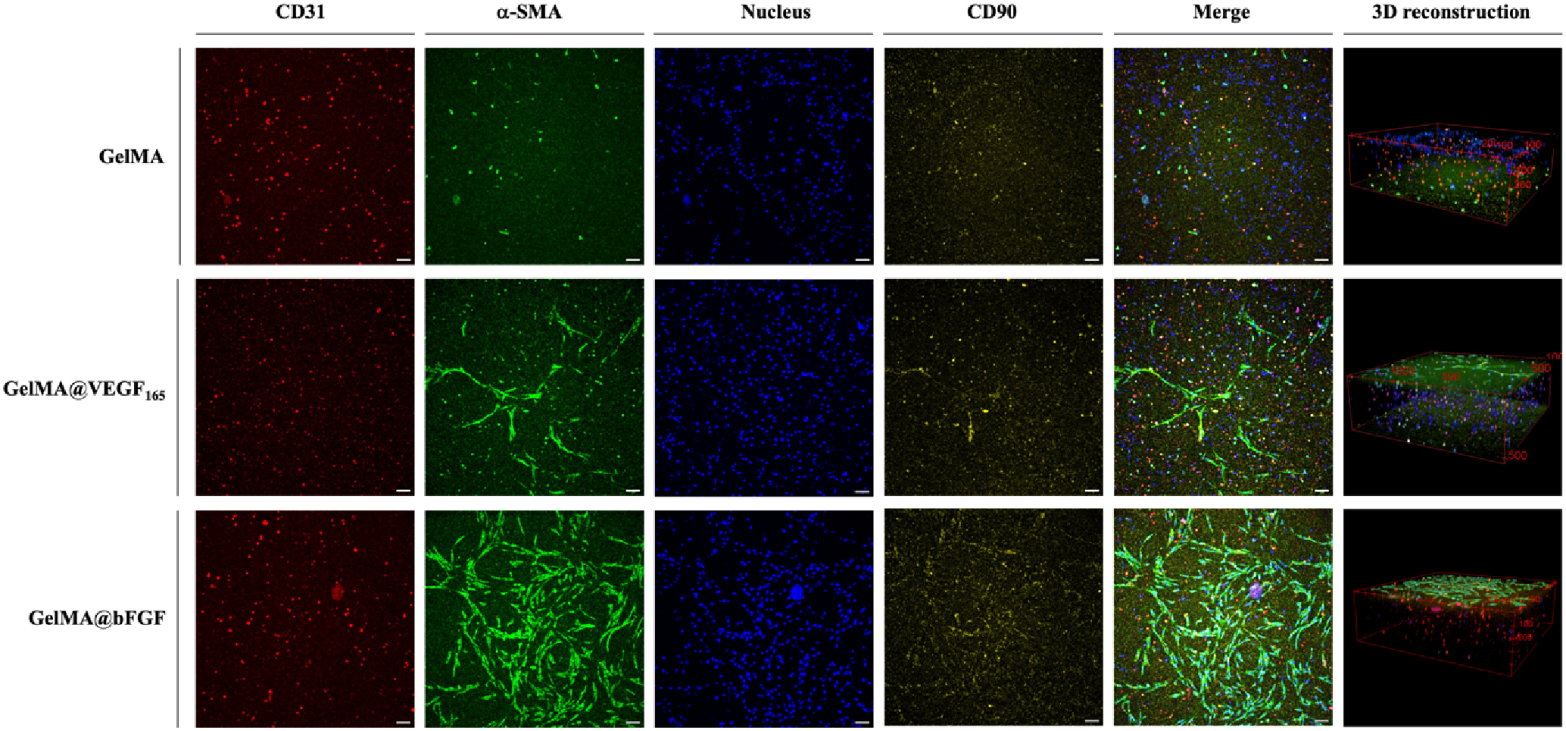
Cell morphology and cell self-organization within the 3D hydrogel cultured in a 50:50 medium on day 7. Confocal live-cell imaging of prestained HUVECs (shown in red, CD31), a smooth muscle marker (shown in green, α-SMA), and hADSCs (shown in red, CD90). The images are displayed in Z-projection and merged channels images. Nuclei counter staining is shown in blue (scale bar: 100 μm).

To identify and visualize encapsulated cells, immunofluorescence staining was employed to label specific markers: CD31 (a HUVEC marker), CD90 (an ADSC marker), and α-SMA (an actin marker). As shown in Figure 5, it is evident that hydrogels with immobilized growth factors exhibit formation of network-like structures within 1 week of culture. Moreover, the cellular reorganization was more apparent in GelMA@bFGF compared to both GelMA@VEGF_165_ and GelMA. The network-like cell reorganization was seen mainly on the surface of the hydrogels. This could be because of the concentration gradient of nutrients in the culture media that stimulates the cell migration toward the surface. However, cells remained in the middle part of the hydrogels, even though the network formation was not clear, and expressed CD31, which confirmed that HUVECs maintained their characteristics. The expression of α-SMA is observed where the cells can spread; thus the signal of α-SMA from within the construct is not pronounced. Additionally, the signal from CD90 was not significant, which might be due to ADSC differentiation, and this marker was no longer expressed after being cocultured with HUVECs in the hydrogels. The enhanced effectiveness of bFGF over VEGF_165_ in promoting cell growth in our coculture system can be attributed to bFGF’s broad mitogenic and angiogenic activities, which influence a variety of cell types beyond endothelial cells, including stem cells and smooth muscle cells. This wide range of action facilitates the promotion of comprehensive tissue growth and regeneration by establishing a favorable environment for the formation and stabilization of vascular structures, unlike VEGF’s more targeted effects on endothelial cells alone.^51^ In a prior study, Cao et al. also revealed that sucrose/aluminum sulfate-based micropellets containing bFGFs promoted higher blood vessel density compared to VEGF-A and VEGF-C within a mouse cornea model.^52^ Based on the observations made, GelMA@bFGF appears to be more effective in promoting network formation and cell growth when compared to GelMA@VEGF_165_.

In this 3D coculturing system, one of the main limitations is that it relies on static *in vitro* culture, which does not accurately replicate the intricate *in vivo* scenario. The presence of concentration gradients of nutrients and oxygen may have played a critical role during the initial phase of vascularization. By implementing a dynamic culture system such as microfluidic chips, it is possible to mitigate the negative effects of these gradients, as well as to better simulate the physiological environment.

## 4 Conclusion

The principal objective of this study is to address the challenges associated with biomimicry and tissue vascularization by examining the impact of grafting vasculogenic-promoting growth factors, VEGF_165_ and bFGF, onto GelMA. The developed methodology entails growth factor conjugations through an EDC/NHS coupling reaction. Our findings indicate that the growth factor-conjugated GelMA hydrogels enhanced the capability of inducing vessel formation *in vitro* in the HUVECs and hADSCs coculture. In particular, GelMA@bFGF exhibited the most pronounced lumen formation and is therefore the most desirable material for vascular tissue engineering applications. The immunorejection should be considered in future studies to maximize the understanding of this material in animal models prior to being further utilized in human. In this study, we suggest that the conjugation of growth factors could be a promising strategy for enhancing vascularization in tissue engineering, which would facilitate other fields of study, including drug discovery, cancer research, stem cell development, and regenerative medicine.

## Supporting Information Available

Additional GelMA and GelMA@GF characterization, GelMA swelling profile, cell proliferation assay, LAP cytotoxicity analysis, and cell imaging data. Figure S1, ^1^H NMR spectrum of GelMA in D_2_O. Figure S2, GelMA@GF characterizations. Figure S3, Water retention of 10%, 15%, and 20% GelMA hydrogels following 24 and 48 hours. Figure S4, CCK-8 cell proliferation assay of coculture of HUVECs and ADSCs on a flat-bottom 96-well plate in different types of media on day 1, 3, and 7. Figure S5, Cytotoxic analysis of different LAP concentrations in conjunction with and without blue light irradiation. Figure S6, Cytotoxicity analysis of different GelMA@GFs concentrations in conjunction with blue light irradiation. Figure S7, Day 3 LIVE/DEAD imaging of encapsulated cells. Figure S8, Day 7 LIVE/DEAD imaging of encapsulated cells (PDF)
