# Supplementary material for: Enhanced Vascular-like Network Formation of Encapsulated HUVECs and ADSCs Coculture in Growth Factors Conjugated GelMA Hydrogels
Source: ACS Biomater Sci Eng. 2024 Apr 18;10(5):3306–15. doi: 10.1021/acsbiomaterials.4c00465 (PMC11094682; doi:10.1021/acsbiomaterials.4c00465)
Supplement: Supplementary file 1 — ab4c00465_si_001.pdf [file ab4c00465_si_001.pdf]

Supplementary Figures

Enhanced Vascular-like Network Formation of Encapsulated HUVECs and ADSCs Co-culture in Growth Factors Conjugated GelMA Hydrogels

Sasinan Bupphathong[1,2,#], Joshua Lim[1,#], Hsu-Wei Fang[2,3,4,#], Hsuan-Ya Tao[1], Chen-En Yeh[5], Tian An Ku[5], Wei Huang[6], Ting Yu Kuo[5], Chih-Hsin Lin [1,]*

1 Graduate Institute of Nanomedicine and Medical Engineering, College of Biomedical Engineering, Taipei Medical University, Taipei 110, Taiwan
2 High-value Biomaterials Research and Commercialization Center, National Taipei University of Technology, Taipei 10608, Taiwan.
3 Department of Chemical Engineering and Biotechnology, National Taipei University of Technology, Taipei 10608, Taiwan
4 Institute of Biomedical Engineering and Nanomedicine, National Health Research Institutes, Zhunan 35053, Taiwan
5 School of Biomedical Engineering, College of Biomedical Engineering, Taipei Medical University, Taipei 110, Taiwan.
6 Department of Orthodontics, Rutgers School of Dental Medicine, Newark, New Jersey, USA.

[#] *Co-first authors*

* Corresponding author: melodylin@tmu.edu.tw

Table of Content

Figure S1. ^{1}H-NMR spectrum of GelMA in D_2O.

Figure S2. GelMA@GF characterizations.

Figure S3. Water retention of 10%, 15%, and 20% GelMA hydrogels following 24 and 48hours.

Figure S4. CCK-8 cell proliferation assay of co-culture of HUVECs and ADSCs on a flat bottom 96-well plate in different types of media on day 1, 3, and 7.

Figure S5. Cytotoxic analysis of different LAP concentrations in conjunction with and without blue light irradiation.

Figure S6. Cytotoxicity analysis of different GelMA@GFs concentrations in conjunction with blue light irradiation.

Figure S7. Day 3 Live/Dead imaging of encapsulated cells.

Figure S8. Day 7 Live/Dead imaging of encapsulated cells.

Supplementary Figures

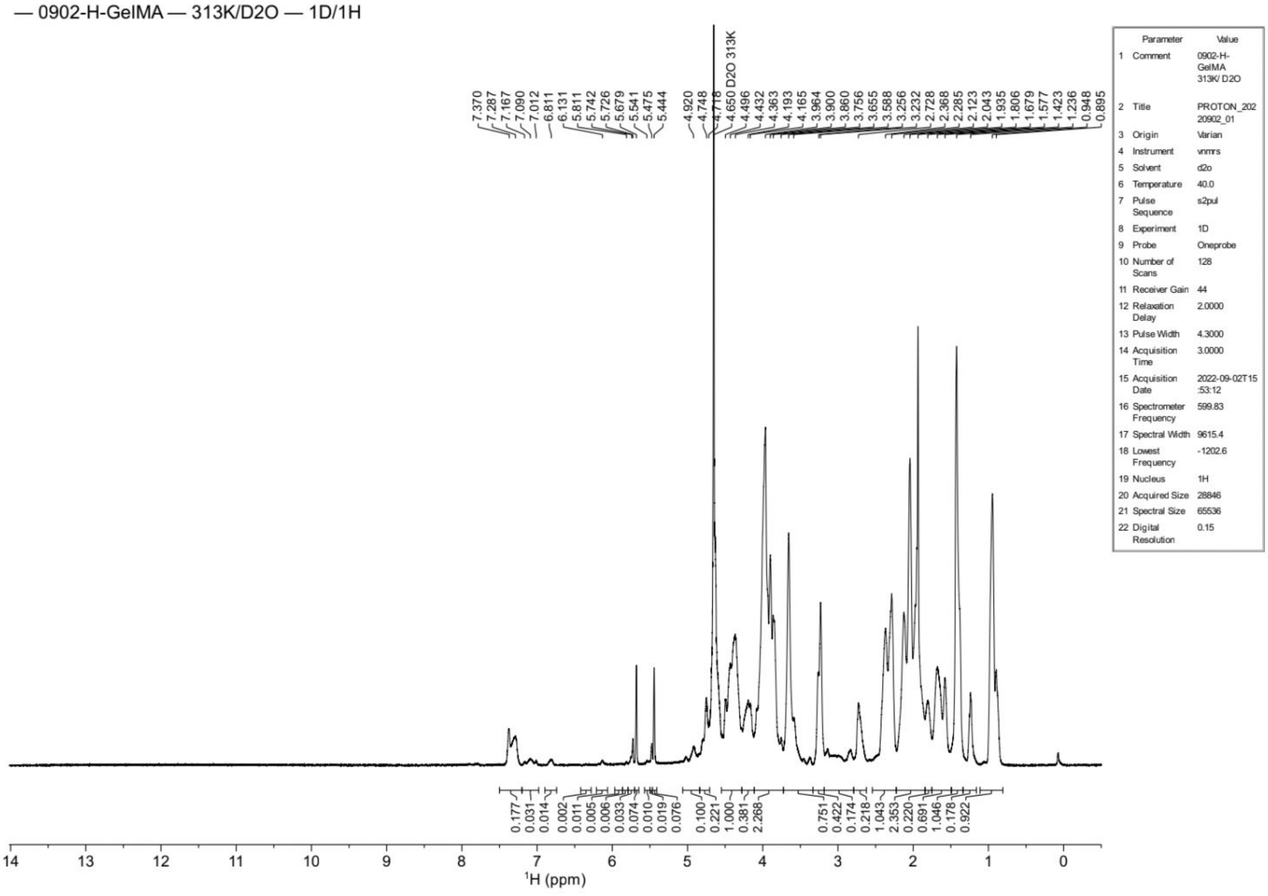

Figure S1. ^{1}H-NMR spectrum of GelMA in D_2O.

Supplementary Figures

A

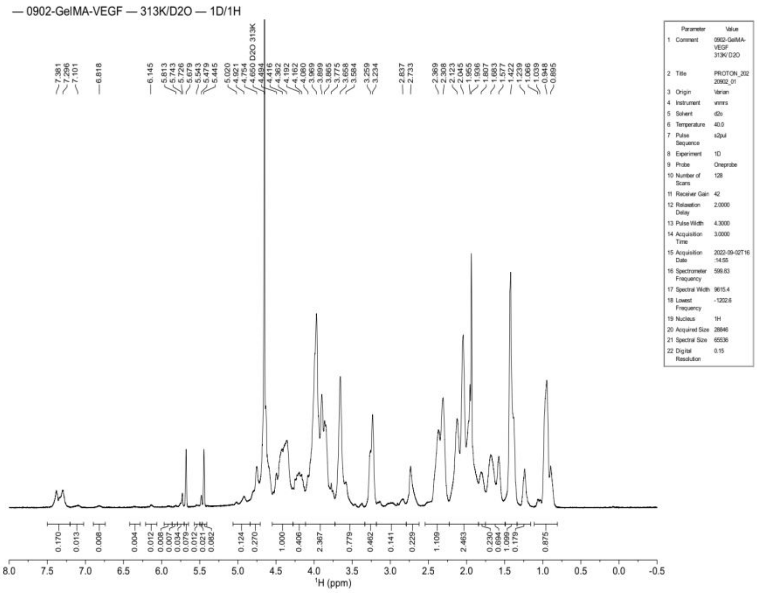

B

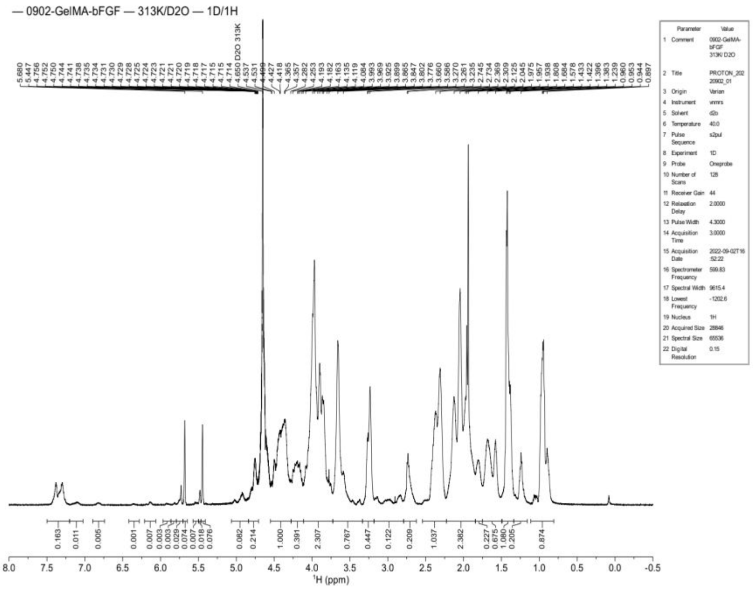

C

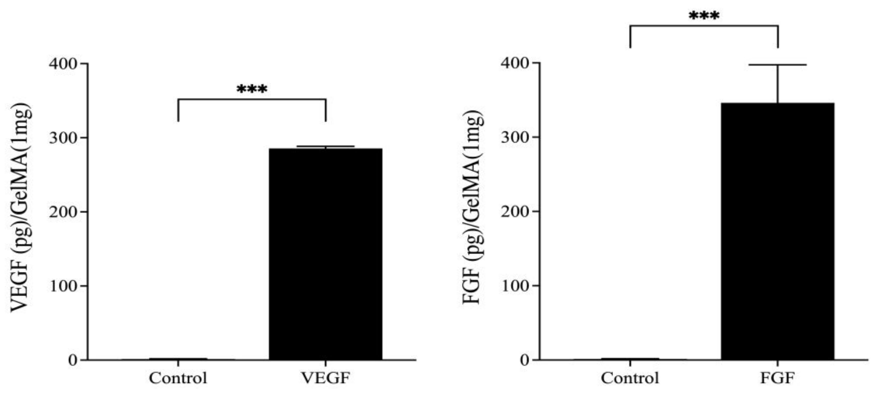

Figure S2. GelMA@GF characterizations (A) ^{1}H-NMR spectrum of GelMA@VEGF$_{165}$ in D_2O and (B) ^{1}H-NMR spectrum of GelMA@bFGF in D_2O. (C) Quantification of bioconjugated VEGF165 and bFGF in GelMA Matrix.

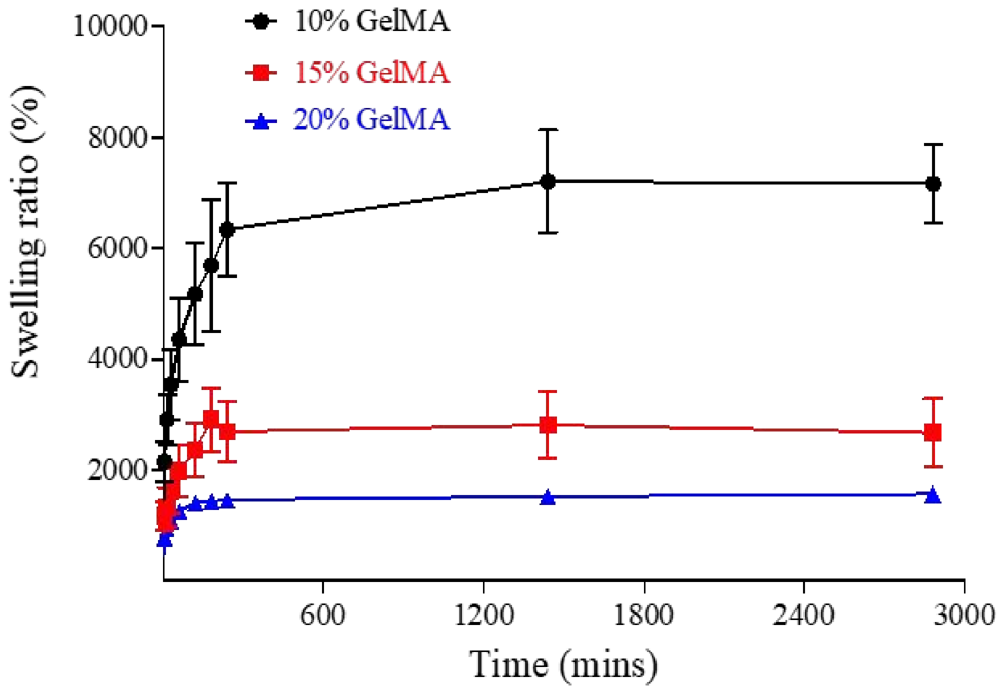

Figure S3. Water retention of 10%, 15%, and 20% GelMA hydrogels following 24 and 48hours.

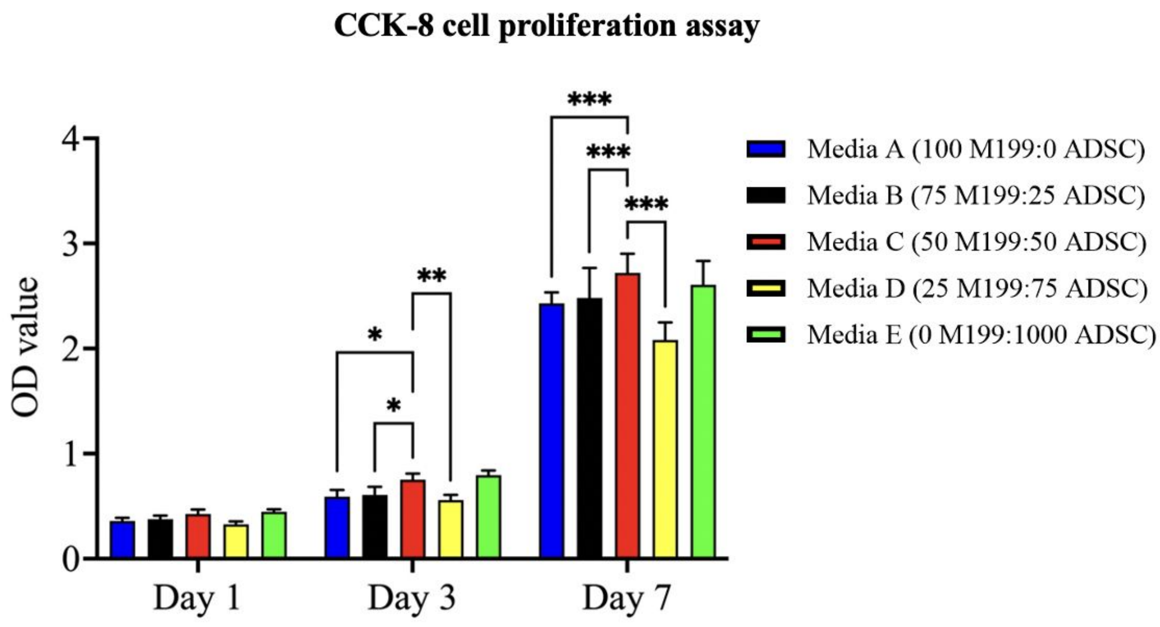

Figure S4. CCK-8 cell proliferation assay of co-culture of HUVECs and ADSCs on a flat bottom 96-well plate in different types of media on day 1, 3, and 7.

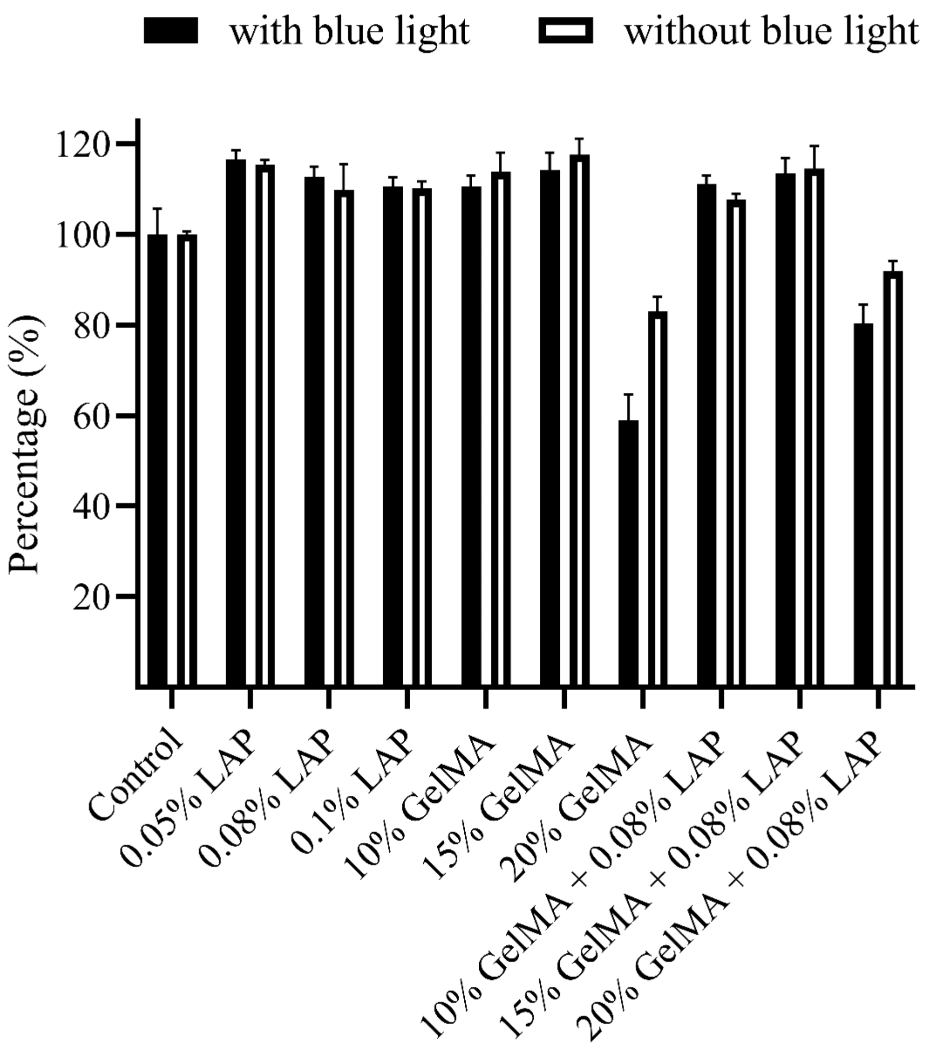

Figure S5. Cytotoxic analysis of different LAP concentrations in conjunction with and without blue light irradiation.

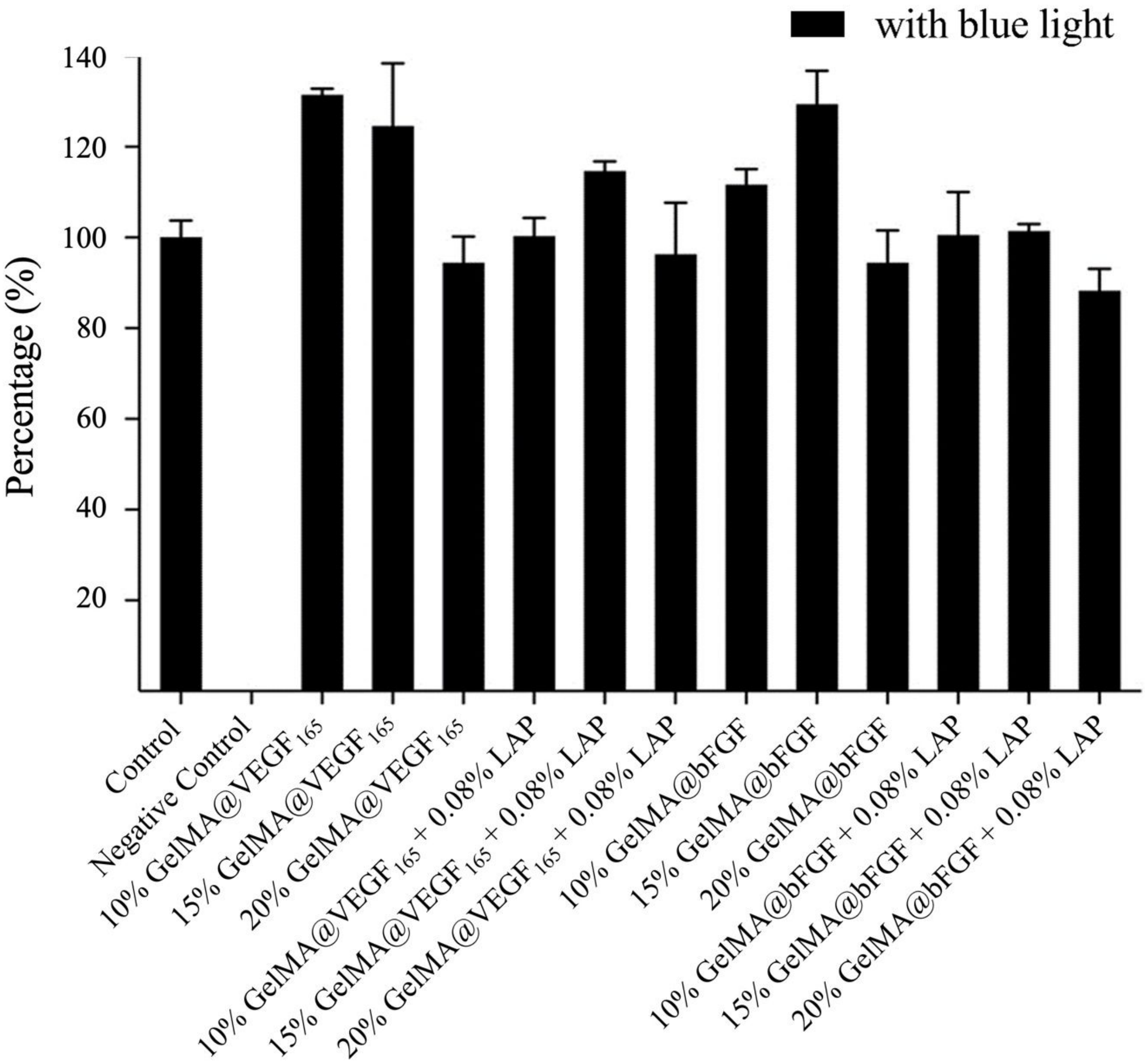

Figure S6. Cytotoxicity analysis of different GelMA@GFs concentrations in conjunction with blue light irradiation.

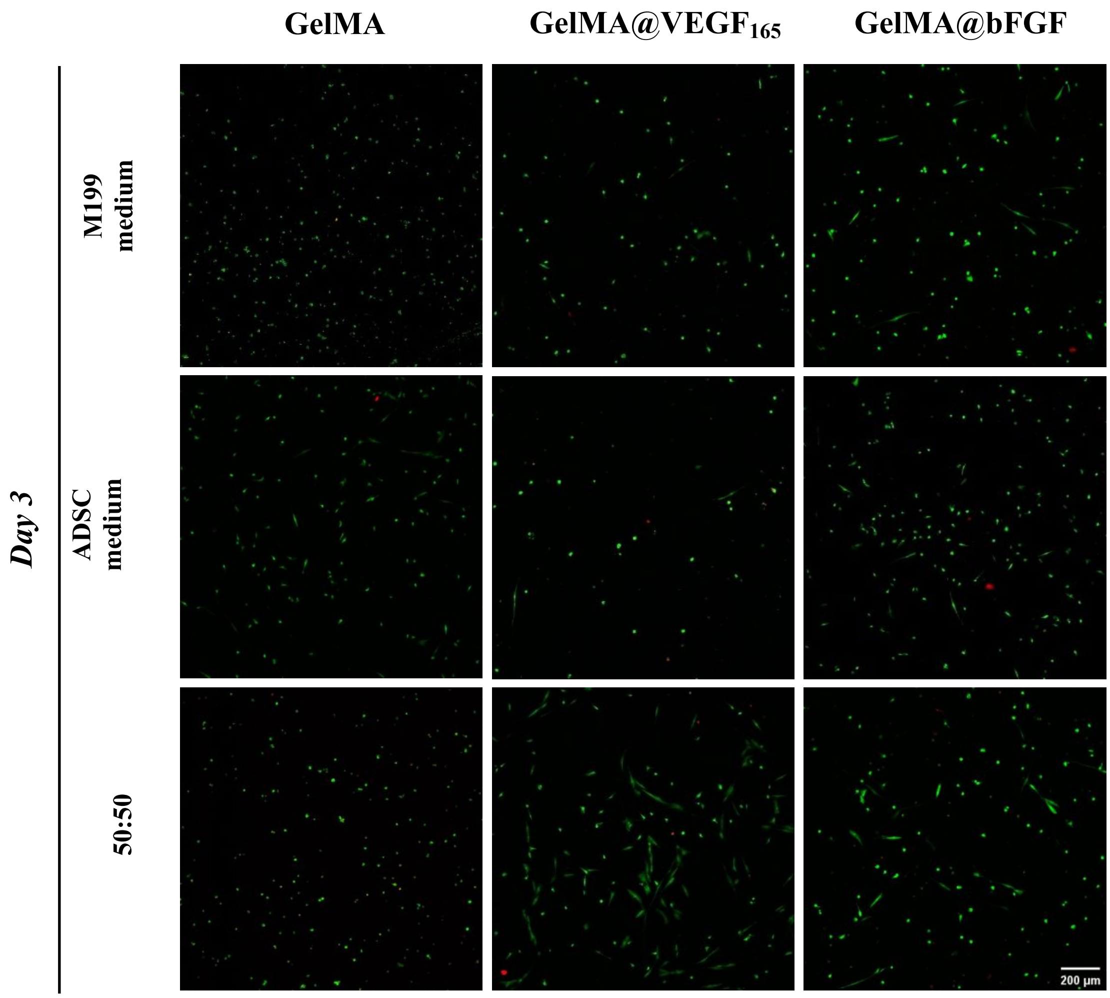

Figure S7. Day 3 Live/Dead imaging of encapsulated cells. The images are displayed in Z-projection and merged channels (Green: live cells, red: dead cells, blue: nuclei, scale bar: 200 μm).

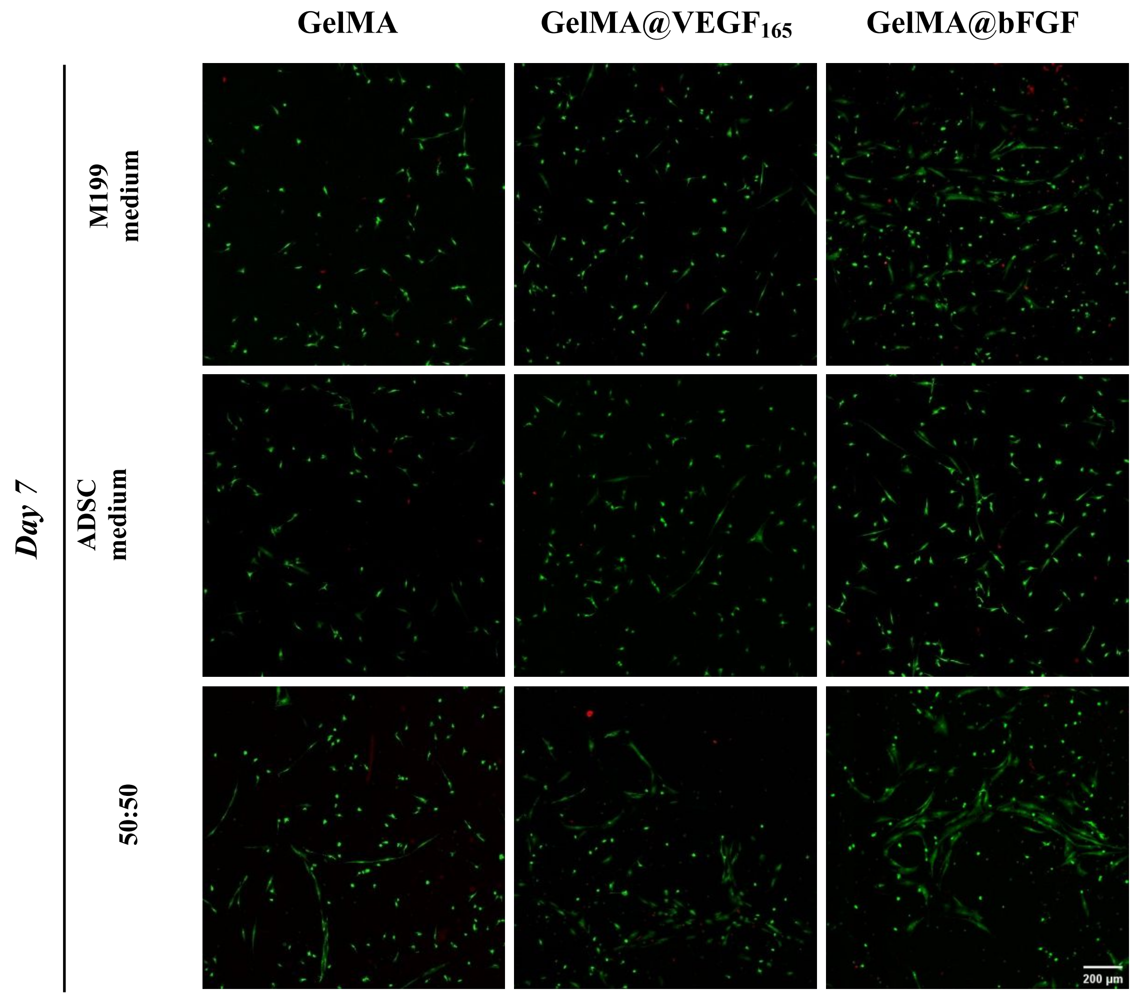

Figure S8. Day 7 Live/Dead imaging of encapsulated cells. The images are displayed in Z-projection and merged channels (Green: live cells, red: dead cells, blue: nuclei, scale bar: 200 µm).
